# Neuromyelitis optica MOG-IgG causes reversible lesions in mouse brain

**DOI:** 10.1186/2051-5960-2-35

**Published:** 2014-03-31

**Authors:** Samira Saadoun, Patrick Waters, Gregory P Owens, Jeffrey L Bennett, Angela Vincent, Marios C Papadopoulos

**Affiliations:** 1 Academic Neurosurgery Unit, St. George’s, University of London, London, UK; 2 Nuffield Department of Clinical Neurosciences, University of Oxford, Oxford, UK; 3 Department of Neurology, University of Colorado Denver, Aurora, Colorado, USA; 4 Department of Ophthalmology, University of Colorado Denver, Aurora, Colorado, USA

**Keywords:** Antibody, Demyelination, Myelin oligodendrocyte glycoprotein, Neuromyelitis optica

## Abstract

**Introduction:**

Antibodies against myelin oligodendrocyte glycoprotein (MOG-IgG) are present in some neuromyelitis optica patients who lack antibodies against aquaporin-4 (AQP4-IgG). The effects of neuromyelitis optica MOG-IgG in the central nervous system have not been investigated in vivo. We microinjected MOG-IgG, obtained from patients with neuromyelitis optica, into mouse brains and compared the results with AQP4-IgG.

**Results:**

MOG-IgG caused myelin changes and altered the expression of axonal proteins that are essential for action potential firing, but did not produce inflammation, axonal loss, neuronal or astrocyte death. These changes were independent of complement and recovered within two weeks. By contrast, AQP4-IgG produced complement-mediated myelin loss, neuronal and astrocyte death with limited recovery at two weeks.

**Conclusions:**

These differences mirror the better outcomes for MOG-IgG compared with AQP4-IgG patients and raise the possibility that MOG-IgG contributes to pathology in some neuromyelitis optica patients.

## Introduction

Most neuromyelitis optica (NMO) patients have IgG against aquaporin-4 (AQP4), here termed AQP4-IgG [1,2]. AQP4 is a water channel protein found in astrocytes throughout the central nervous system (CNS), especially in perivascular astrocyte foot processes [3]. In cultured cells, AQP4-IgG binds extracellular conformational domains of AQP4 and activates complement, causing cell lysis [4]. In mice, intracerebral injection of AQP4-IgG activates co-injected human complement (C_hu_) and damages the astrocytes [5,6]. Inflammatory cells then enter the lesion causing further tissue injury including demyelination and axonal damage. In AQP4-IgG NMO patients, recovery after an attack is usually limited [7-9].

A few NMO patients without AQP4-IgG have IgG against myelin oligodendrocyte glycoprotein (MOG-IgG), which recognize extracellular conformational domains of MOG [10-13]. MOG is expressed on the outer surface of CNS myelin sheaths and comprises about 0.05% of total myelin protein [14]. There is mounting evidence that MOG-IgG NMO has more favorable clinical outcome than AQP4-IgG NMO, with resolution of imaging abnormalities [10,11,15,16]. It is currently unclear whether MOG-IgG plays any role in NMO by causing lesions in the CNS *in vivo*. Here we compared the effects of MOG-IgG with those of AQP4-IgG in the intracerebral injection mouse model. We used total IgG from a normal subject (IgG_CON_) and from NMO patients with AQP4-IgG (IgG_AQP4_) or MOG-IgG (IgG_MOG_).

## Materials and methods

### IgG and complement

NMO patients with MOG-IgG or AQP4-IgG were identified using live cell-based assays. Briefly, AQP4-IgG and MOG-IgG positivity was determined by visualization of binding to human embryonic kidney cells, transfected with the extracellular and trans-membrane domains of MOG or with full-length M23-AQP4. Details of the assays are given elsewhere [5,10,11,15]. IgG was purified using Protein G from sera or plasmas of five patients with AQP4-IgG NMO, five MOG-IgG NMO or one healthy volunteer. The effect of injecting IgG and C_hu_ from healthy volunteers into mouse brain was extensively investigated in our earlier studies [5,17,18]. The purified, dialysed and pooled total IgG preparations (6 – 38 mg/ml) are termed IgG_AQP4_, IgG_MOG_ and IgG_CON_. Clinical details of the 5 AQP4-IgG + [5] and 5 MOG-IgG + [11,15] patients are given elsewhere. To deplete MOG-IgG, the IgG_MOG_ was adsorbed by incubation with MOG-HEK cells until MOG-IgG became undetectable (IgG_MOG(AdsMOG-HEK)_). IgG_MOG_ adsorbed against untransfected HEK cells (IgG_MOG(AdsHEK)_) was used as control. A chimeric mouse-human recombinant monoclonal anti-mouse MOG antibody, MOG-IgG_2B7_, was produced as described [19]. Human recombinant monoclonal anti-AQP4 IgG_1_, termed AQP4-IgG_53_, was also generated [20]. A measles virus-specific antibody termed CON-IgG_2B4_ was used as isotype control [20]. The source of C_hu_ was fresh serum from healthy volunteers [5].

### Mice

Experiments were performed at St. George’s, University of London using CD1 mice 8 – 12 30 – 35 g, 8 – 12w old. Protocols were approved by the British Home Office (Project Licence, PPL 70/7081). After administering 2,2,2-tribromoethanol i.p., mice were mounted onto a stereotactic frame (Benchmark, Neurolab, St Louis, MO, USA). Four burrholes were made on the right side using a high speed drill (0.7 mm burr, Foredom, Bethel, CT, USA) at the following coordinates in millimetres from the bregma (lateral, anterior): (1, 0), (1, −1), (1, −2), (2, −1). Mice were allocated to the different experimental groups by a person unaware of the aim of the study. A 30 g needle attached to 50 ml gas-tight glass syringe (Hamilton, Reno, NV, USA) was inserted 3 mm deep to micro-infuse (1 μL/min) into the right hemisphere 16.8 μL IgG_MOG_, IgG_AQP4_ or IgG_CON_ or 16.8 μL (20 μg) MOG-IgG_2B7_ or AQP4-IgG_53_ + 11.2 μL C_hu_ (or normal saline) as described [5]. Rectal temperature was kept 37 – 38°C with a heating lamp. After regaining the righting reflex, mice were returned to their cages, kept in 12 hour light/dark cycle and given water and normal chow *ad libitum*. Mice (5 per group) were killed at 24 hours, seven days or two weeks. Investigators were unaware of which antibody was injected.

### Mouse brain histology and immunohistology

Mice were anaesthetized and perfused-fixed by injecting 4% formaldehyde through the left cardiac ventricle. Brains were removed, post-fixed in 4% formaldehyde overnight and processed into paraffin. Coronal tissue sections (7 μm thick) through the injection tract were stained with H + E, Luxol Fast Blue (LFB) [5] or immunostained.

For diaminobenzidine immunostaining, the sections were unmasked in citrate, incubated with primary antibody (one hour, 25°C), biotinylated secondary antibody (1:500, one hour, 25°C) and visualized using the Vectastain HRP kit (Vector Labs, Peterborough, UK). We counterstained nuclei with haematoxylin. Primary antibodies were rabbit anti-AQP4 (1:100), rabbit anti-glial fibrillary acidic protein (GFAP, 1:200), mouse anti-NeuN (1:200), (Millipore, Livingstone, UK), mouse anti-myelin basic protein (MBP, 1:400, Leica, Newcastle, UK), mouse anti-neurofilament-70 (1:600, DAKO, Ely, UK), rabbit anti-C5b-9 (1:100, Abcam, Cambridge, UK) and rat anti-CD45 (1:200, BD Bioscience, Oxford, UK). Samples were then incubated with the appropriate species biotinylated secondary antibody (1:500, Vector Laboratories). Immunostaining was visualized brown using the Vectastain horseradish peroxidase kit (Vector Laboratories) followed by diaminobenzidine/H_2_O_2_. Nuclei were counterstained blue with haematoxylin.

For immunofluorescence staining, we used rabbit anti-Ankyrin G (AnkG, InsightBio, Wembley, UK) or rabbit anti-Contactin associated protein (Caspr) from Abcam (1:200, 12 hours, 25°C) followed by Alexafluor-linked anti-rabbit antibody (1:200, one hour, 25°C, Invitrogen, Paisley, UK).

To determine MOG-IgG binding to mouse brain sections, brains were removed, immersed in 30% sucrose overnight, embedded in OCT and cut into 7 μm sections. These were fixed in acetone and exposed to IgG_MOG(AdsHEK)_, IgG_MOG(AdsMOG-HEK)_, IgG_CON_ (1:100) or MOG-IgG_2b7_ (1 mg/mL) ± rabbit anti-MOG (1:100, InsightBio) for one hour at 25°C followed by Alexafluor-linked anti-human ± anti-rabbit IgG (1:200, one hour, 25°C, Invitrogen) and DAPI.

Photomicrographs were taken using an Olympus BX-51 microscope.

### Data analysis

Coded photomicrographs were analysed with ImageJ (v1.45S, NIH). Neurofilament immunoreactivity in the injected hemisphere was quantified as mean staining intensity minus background. AnkG and Caspr expression was the number of fluorescent spots/mm^2^ in four photomicrographs, 90 μm × 67 μm, taken from the injected hemisphere 0.5 mm from the needle tract. After subtracting background, formatting images to 8-bit, adjusting threshold, the ‘analyse particles’ function of Image J was used. Spots < 0.01 μm^2^ were excluded as noise.

### Statistics

Data are mean ± standard error. We used Student t-test or ANOVA with Student-Newman-Keuls *post-hoc* analysis. Significance is *P* < 0.05*, 0.01**, 0.001***.

## Results

### Lesions induced by IgG_MOG_ compared to IgG_AQP4_

**Figure 1:**
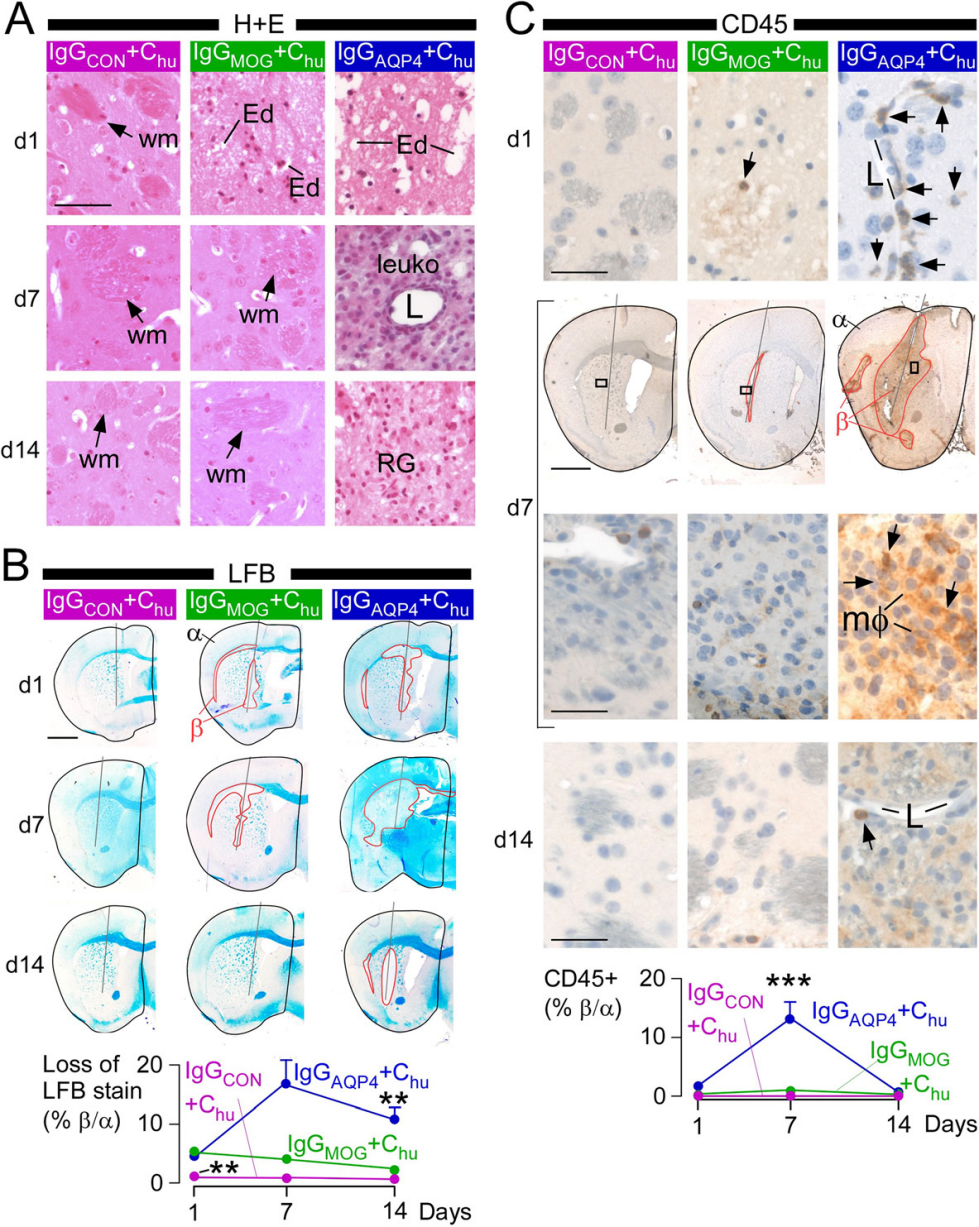
**Brain lesions caused by MOG-IgG and AQP4-IgG.** Mice received IgG_CON_ + C_hu_ (purple), IgG_MOG_ + C_hu_ (green) or IgG_AQP4_ + C_hu_ (blue), were killed at 24 hours (d1), seven days (d7) or two weeks (d14) and coronal brain sections were cut through the injection site. **A**. H + E staining. Ed, edema; L, lumen; leuko, leukocytes; RG, reactive glia; wm, white matter. **B**. (*Top*) LFB staining. Red line, loss of LFB staining. (*Bottom*) % Loss of LFB stain (area without LFB/ipsilateral hemispheric area) *vs.* days since injection. **C**. (*Top*) CD45 immunostain. Each boxed area in d7 top is shown magnified below. Arrows, CD45+ cells; L, vessel lumen; mϕ, macrophages. (*Bottom*) % CD45+ area (CD45+ area/ipsilateral hemispheric area) *vs.* days since injection. Mean ± SEM, 5 mice per group. P < 0.01**, 0.001*** (compared with each of the other two groups). Bar 50 μm **(A)**, 1 mm (**B**, **C** d7 top), 20 μm (**C** d7 bottom).

IgG_MOG_ + C_hu_ caused brain edema at 24 hours, but by seven days and two weeks the brain appeared normal (Figure 1A). Although IgG_AQP4_ + C_hu_ also caused edema at 24 hours, at seven days there was marked leukocyte infiltration and by two weeks reactive gliosis (Figure 1A). IgG_MOG_ + C_hu_ caused loss of Luxol Fast Blue (LFB) staining at 24 hours, but this had reversed by two weeks, while the IgG_AQP4_ + C_hu_ – injected tissue showed increased loss of LFB staining at seven days and only partially recovered at two weeks (Figure 1B). The recruitment of inflammatory cells also differed markedly between the two preparations. IgG_MOG_ + C_hu_ did not produce inflammation while IgG_AQP4_ + C_hu_ caused inflammation at 24 hours (perivascular neutrophils) and seven days (mostly macrophages) (Figure 1C).

**Figure 2:**
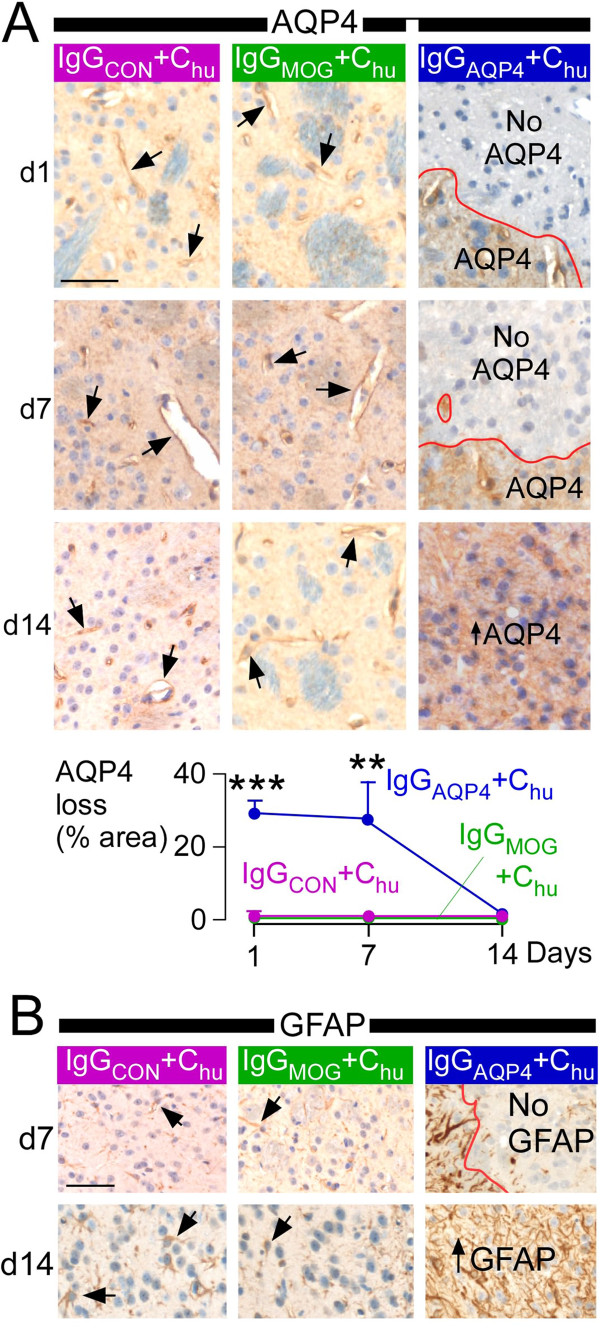
**Effect of MOG-IgG and AQP4-IgG on astrocytes.** Mice received IgG_CON_ + C_hu_ (purple), IgG_MOG_ + C_hu_ (green) or IgG_AQP4_ + C_hu_ (blue), were killed at 24 hours (d1), seven days (d7) or two weeks (d14) and coronal brain sections were cut through the injection site. **A**. (*Top*) AQP4 immunostain. Arrows, perivascular immunostain; red line, lesion border; ↑AQP4, area with high AQP4 (reactive astrocytes). (*Bottom*) % AQP4 loss (AQP4 immunonegative area/ipsilateral hemispheric area) *vs.* days since injection. **B**. GFAP immunostain. Arrows, GFAP+ processes; red line, lesion border; ↑GFAP, area with high GFAP (reactive astrocytes). Mean ± SEM, 5 mice per group. P < 0.01**, 0.001*** (compared with each of the other two groups). Bar 50 μm **(A, B)**.

We immunostained for two astrocyte markers, AQP4 and GFAP. Loss of AQP4 and GFAP was seen in IgG_AQP4_ + C_hu_ – injected brains (at 24 hours and seven days) but IgG_MOG_ + C_hu_ did not reduce AQP4 and GFAP (Figure 2). At two weeks there was marked gliosis (increased AQP4 and GFAP) in brains injected with IgG_AQP4_ + C_hu_, compared to little gliosis in brains that received IgG_MOG_ + C_hu_ (Figure 2).

### MOG-IgG binds mouse MOG and causes loss of LFB staining

**Figure 3:**
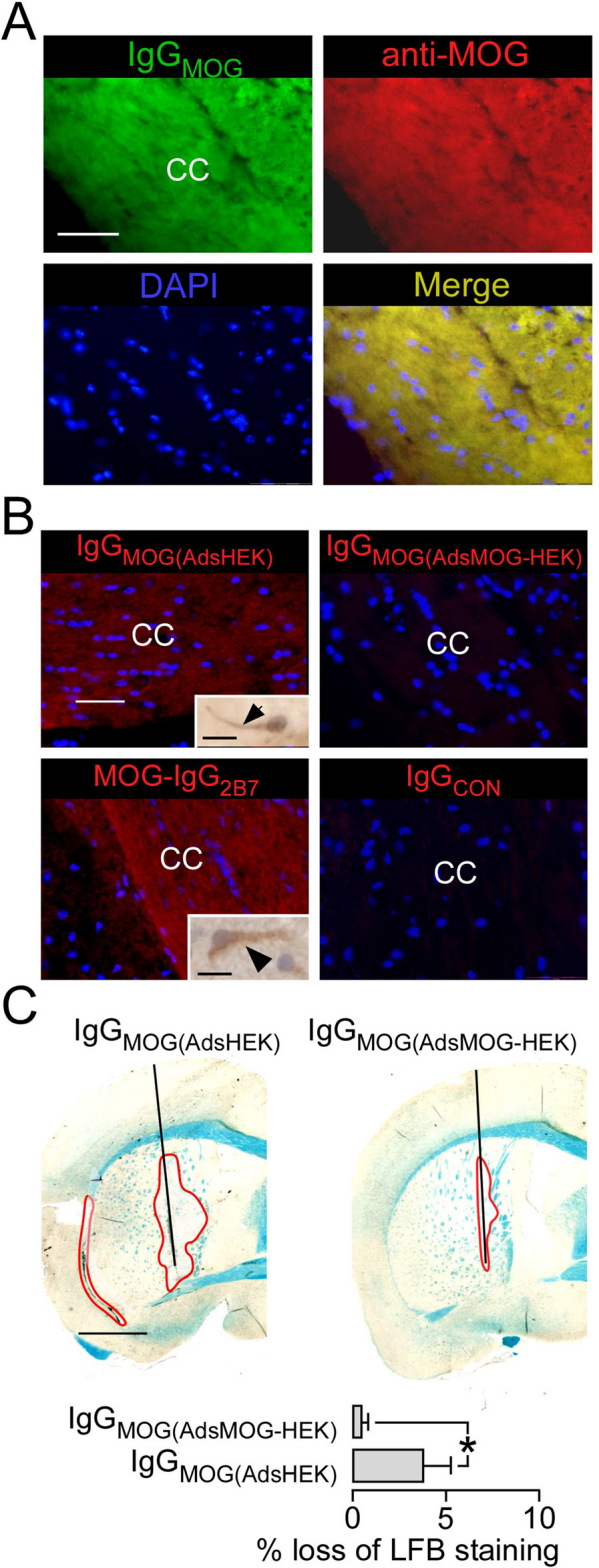
**MOG-IgG binds mouse MOG and causes loss of LFB staining. A**. Mouse corpus callosum (CC) fluorescently immunostained with IgG_MOG_ (green) and anti-MOG (red). DAPI nuclear stain. **B**. CC immunostained fluorescent red with IgG_MOG_, IgG_MOG(AdsMOG-HEK)_, MOG-IgG_2B7_ and IgG_CON_. DAPI nuclear stain. Insets – immunopositive (DAB) gray matter neurons. **C**. Mouse brain injected with IgG_MOG(AdsHEK)_ + C_hu_ or IgG_MOG(AdsMOG-HEK)_ + C_hu_. (*Top*) LFB stained sections at 24 hours. Red line, no LFB staining. (*Bottom*). Data summary. 5 mice per group. Mean ± SEM. P < 0.05*. Bar 10 μm (**A** insets), 50 μm **(A, B)**, 1 mm **(C)**.

To confirm that IgG_MOG_ binds mouse myelin, it was applied to brain sections. IgG_MOG_ bound the corpus callosum; binding co-localized with a commercial anti-MOG antibody (Figure 3A). IgG_MOG_ adsorbed by incubation with MOG-expressing human embryonic kidney (MOG-HEK) cells until MOG-IgG became undetectable (IgG_MOG(AdsMOG-HEK)_) did not bind the corpus callosum, unlike IgG_MOG_ adsorbed against untransfected HEK cells (IgG_MOG(AdsHEK)_) (Figure 3B). To confirm that the MOG-IgG was responsible for the loss of LFB staining, the two adsorbed preparations were injected with C_hu_ and mice were killed at seven days. Loss of LFB staining in the injected hemisphere was only found when IgG_MOG(AdsHEK)_ + C_hu_ was used (Figure 3C).

### MOG-IgG_2B7_ causes loss of LFB staining largely independent of immune cells or complement activation

**Figure 4:**
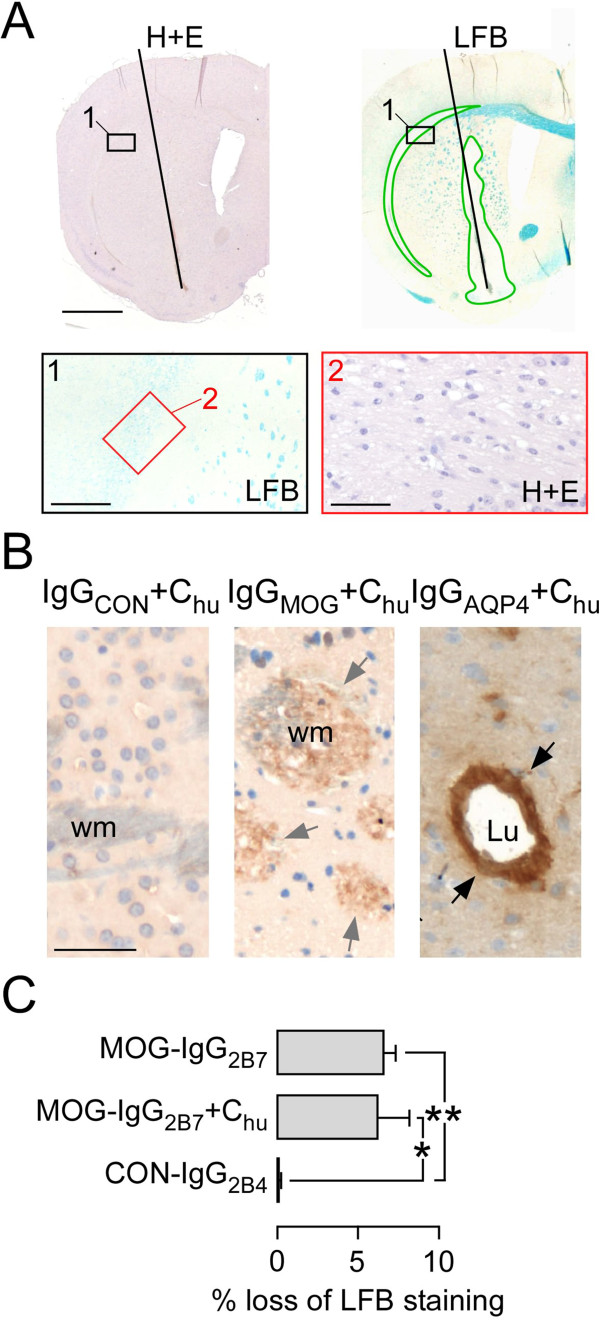
**MOG-IgG**_**2B7 **_**causes loss of LFB staining largely independent of immune cells or complement activation. A**. Adjacent sections stained with H + E and LFB at seven days after injecting MOG-IgG_2B7_ + C_hu_. Green line shows loss of LFB staining. Rectangles show sites of Sections. **B**. Mouse brain immunostaned for C5b-9 at 24 hours after injection of IgG_CON_ + C_hu_, IgG_MOG_ + C_hu_, or IgG_AQP4_ + C_hu_. Lu, lumen; wm, white matter. Weak (gray arrows) and strong (black arrows) immunoreactivity. **C**. Loss of LFB staining at 24 hours after injection of MOG-IgG_2B7_, MOG-IgG_2B7_ + C_hu_, or isotype control (CON-IgG_2B4_). 5 mice per group. Mean ± SEM. P < 0.05*, < 0.01**. Bar 50 μm (**A** bottom right, **B**), 200 μm (**A** bottom left), 1 mm (**A** top).

In case the amount of MOG-IgG in the patient preparations was insufficient to cause inflammatory cell infiltration, a large amount (20 μg) of the humanized anti-mouse MOG-IgG_2B7_ was co-injected with C_hu_. At seven days, MOG-IgG_2B7_ + C_hu_ caused loss of LFB staining, but without inflammatory cell infiltration (Figure 4A). At 24 hours after injecting MOG-IgG_2B7_ + C_hu_ there was faint C5b-9 immunoreactivity in white matter tracts suggesting slight complement activation, whereas injection of a monoclonal recombinant anti-AQP4 (AQP4-IgG_53_) + C_hu_ caused strong perivascular C5b-9 immunoreactivity (Figure 4B). Moreover, intracerebral injection of MOG-IgG_2B7_ without C_hu_ produced loss of LFB staining at 24 hours similar to MOG-IgG_2B7_ + C_hu_ (Figure 4C).

### MOG-IgG causes reversible damage to myelinated axons

**Figure 5:**
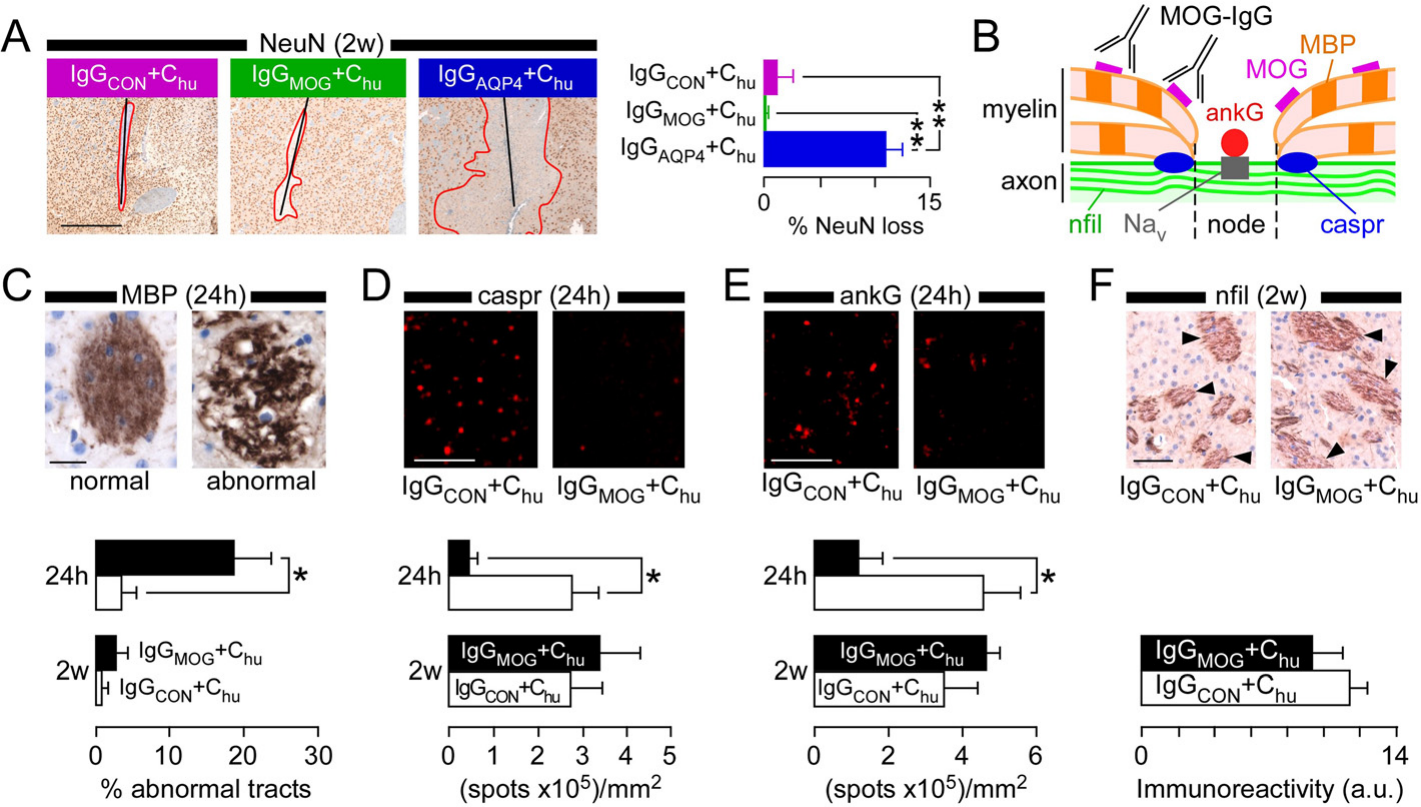
**Effect of MOG-IgG on neurons. A**. (*Left*) NeuN immunoreactivity at 2w. Area lacking neurons outlined red. (*Right*) % NeuN loss (NeuN immunonegative area/ipsilatral hemispheric area). **B**. Node of Ranvier: ankG, ankyrin G; Caspr, contactin associated protein; MBP, myelin basic protein; MOG, myelin oligodendrocyte glycoprotein; Na_v_, voltage-gated Na^+^ channel; Nfil, neurofilament. **C**. MBP immunoreactivity. (*Top*) Normal and abnormal white matter tracts. (*Bottom*) % abnormal tracts in injected hemisphere. **D**. Caspr immunoreactivity within white matter tracts. (*Top*) Hemispheres injected with IgG_CON_ + C_hu_ and IgG_MOG_ + C_hu_. (*Bottom*) Data summary. **E**. AnkG immunoreactivity within white matter tracts. (*Top*) Hemispheres injected with IgG_CON_ + C_hu_ and IgG_MOG_ + C_hu_. (*Bottom*) Data summary. **F**. Nfil immunoreactivity. (*Top*) Hemispheres injected with IgG_CON_ + C_hu_ and IgG_MOG_ + C_hu_. Arrowheads: white matter tracts. (*Bottom*) Data summary (a.u. arbitrary units). 5 mice per group. P <0.05*, <0.01**. Bar 0.5 mm **(A)**, 20 μm **(C)**, 10 μm **(D, E)**, 50 μm **(F)**.

At two weeks there was marked neuronal loss in IgG_AQP4_ + C_hu_ lesions compared to little neuronal loss in brains injected with IgG_MOG_ + C_hu_ (Figure 5A). We investigated the effect of IgG_MOG_ + C_hu_ on myelin and axonal proteins including myelin basic protein (MBP), neurofilament, ankyrin G (AnkG) and contactin associated protein (Caspr) (Figure 5B). MBP adheres adjacent cytoplasmic faces of myelin together, neurofilament provides structural support for axons, AnkG clusters voltage-gated Na^+^ channels at nodes of Ranvier [21] and Caspr attaches paranodal myelin loops to the axons [22]. At 24 hours after IgG_MOG_ + C_hu_ injection, MBP expression appeared abnormal (Figure 5C) and there was significant reduction in AnkG (Figure 5D) and Caspr (Figure 5E) immunoreactivities. At two weeks, the MOG-IgG + C_hu_ – induced changes in MBP, AnkG and Caspr had recovered and neurofilament expression was normal (Figure 5F), indicating intact axons.

## Discussion

Although there is growing interest in the potential pathogenicity of MOG antibodies in NMO, the effects of NMO MOG-IgG have not been explored *in vivo*. Our results indicate that MOG-IgG directly damages myelin. The detrimental effects of MOG-IgG markedly differ from those of AQP4-IgG and are reversible (see Table 1).

**Table 1:** Comparison of MOG-IgG with AQP4-IgG lesions in mouse brain

**Characteristic**	**MOG-IgG**	**AQP4-IgG**
**Target cell**	Oligodendrocyte (myelin)	Astrocyte (foot process)
**Lesion onset**	Within hours of exposure to MOG-IgG	Within hours of exposure to AQP4-IgG
**Effect on astrocytes**	No major effect (normal AQP4 and GFAP)	Astrocyte death (loss of AQP4 and GFAP)
**Effect on neurons**	No major effect	Neuronal death (loss of NeuN, FJ-C staining [5])
**Effect on oligodendrocytes**	Change in myelin (loss of LFB)	Loss of myelin (loss of LFB)
**Effect on axons**	Myelin (transient change in MBP)	Permanent loss of myelin
	Intact axons (normal nfil)	Axonal degeneration (β-APP expression [5])
	Node of Ranvier (transient change in casp and ankG)	
**Inflammatory cell infiltration**	No	Yes
**Complement activation**	Slight (in white matter tracts)	Marked (perivascular)
	Not required for lesion to develop	Essential for lesion to develop
**Recovery**	Yes, within 2 weeks	No, pan-necrosis followed by glial scarring

β-APP, beta amyloid precursor protein; FJ-C, fluorojade C; GFAP, glial fibrillary acidic protein; LFB, Luxol fast blue; MBP, myelin basic protein; NeuN, neuronal nuclear marker; nfil, neurofilament.

AQP4-IgG lesions are characterized by astrocyte damage followed by leukocyte infiltration that entirely depend on complement activation [5]. We showed that recovery of myelin loss in AQP4-IgG lesions is limited, with gliosis and neuronal death. This finding may explain why clinical recovery after AQP4-IgG NMO attacks is often limited [7-9]. By contrast, MOG-IgG, as examined here, damages myelin and axons temporarily, with little complement activation, and no leukocyte infiltration. The myelin and axonal recovery and lack of neuronal death mirror the reported good outcomes of MOG-IgG NMO patients [10,11,15,16].

One study suggested that IgG_MOG_ obtained from children with demyelination does not bind mouse MOG [23], but another study showed that human MOG-IgG binds mouse MOG [24]. Our IgG_MOG_ samples obtained from adult NMO patients, and the anti-mouse MOG-specific monoclonal antibody, both recognized mouse MOG in frozen brain sections, and produced comparable LFB loss without inflammation. This discrepancy may be due to differences in MOG-IgG levels and specificity or differences in MOG glycosylation state, which plays a key role in MOG-IgG binding [24], between children and adults.

The effects of MOG-IgG on cultured oligodendrocytes have already been studied. MOG-IgG binds extracellular epitopes on MOG [23] and can cause crosslinking [25] and internalization [12] of MOG molecules and reversible retraction of oligodendrocyte processes [25]. At high concentration, MOG-IgG causes complement-mediated lysis of MOG-expressing cells [12]. Passive transfer of MOG-IgG antibodies exacerbates CNS damage in experimental autoimmune encephalomyelitis rodent models in which cellular immunity is the predominant pathogenic mechanism [26,27]. Using the intracerebral injection mouse model, we have shown unequivocally that NMO MOG-IgG directly damages myelin *in vivo* independent of pre-existing cellular immunity and complement.

MOG-IgG changed MBP architecture and reduced expression of axonal proteins. Caspr and AnkG are required for the integrity of the nodes of Ranvier and normal action potential firing [21,22]. Mice that lack MBP have a characteristic motor dysfunction including tremor and seizures [28], mice that lack Caspr have severe motor paresis [22] whereas mice lacking cerebellar ankG develop progressive ataxia [21]. Therefore, the altered MBP expression and reduced Caspr and AnkG expression produced by MOG-IgG are predicted to produce a neurological deficit if the NMO lesion is in an eloquent region of the CNS. Unlike AQP4-IgG, MOG-IgG did not produce axonal disintegration or neuronal death. Given the 96% homology between mouse and human MOG [14], our findings raise the possibility that MOG-IgG may also cause similar reversible lesions in the human CNS.

MOG-IgG has been reported in other non-NMO diseases including multiple sclerosis, acute disseminated encephalomyelitis and even some normal subjects [29]. Does MOG-IgG from these non-NMO subjects also cause the same reversible CNS changes, as described here for NMO MOG-IgG? This question is difficult to answer at present because of the variety of assays used to detect MOG-IgG. For example, the assay used here, which employs C-terminal truncated rather than full-length MOG, did not detect MOG-IgG in adult multiple sclerosis patients and normal individuals [11], which suggests that different assays detect different subpopulations of MOG-IgG. It is important to first standardize the assays before determining which subpopulations of MOG-IgG can cause CNS damage and in which diseases.

The mechanism of MOG-IgG-induced myelin damage *in vivo* is unknown. Our data show that MOG-IgG – mediated myelin damage is a direct effect of MOG-IgG and that complement activation is not necessary. MOG-IgG binding may cause MOG conformational changes or internalization that disrupts the myelin structure and secondarily alters axonal protein expression. To explain the lack of complement involvement, we hypothesize that, after MOG-IgG binding, MOG might not aggregate (because of its low abundance) or MOG might become internalized (thus prohibiting C1q activation). The full recovery within two weeks of the MOG-IgG-induced LFB, MBP, ankG and Caspr changes suggests that MOG-IgG does not kill the oligodendrocytes, but causes a reversible damage.

Our findings raise the possibility that MOG-IgG contributes to pathology in some NMO patients. If MOG-IgG is pathogenic, antibody depletion (plasmapheresis) or suppression with steroids should be effective, as indeed appears to be the case [10,11,15,16]. Conversely, some of the newly proposed therapies for AQP4-IgG NMO, such as sivelestat for inhibiting neutrophils [17], or eculizumab for inhibiting complement [30], are less likely to be needed in MOG-IgG NMO. Examining lesions from MOG-IgG NMO patients may help elucidate the pathogenicity of MOG-IgG in the human CNS.

## Conclusions

MOG-IgG obtained from neuromyelitis optica patients causes myelin changes and alters the expression of axonal proteins when injected in mouse brain. These effects are not associated with inflammatory cell infiltration, are largely independent of complement and recover within two weeks. AQP4-IgG obtained from neuromyelitis optica patients causes complement-mediated myelin loss, inflammatory cell infiltration, neuronal and astrocyte death with limited recovery at two weeks. These findings raise the possibility that MOG-IgG contributes to pathology in some neuromyelitis optica patients.

### Availability of supporting data

No supporting data.

## Abbreviations

AnkG: Ankyrin G; AQP4: Aquaporin-4; AQP4-IgG: Aquaporin-4 IgG found in most neuromyelitis optica patients; C5b-9: Complement membrane attack complex; Caspr: Contactin associated protein; Chu: Human complement; CNS: Central nervous system; CON-IgG2B4: Monoclonal (2B4) control IgG; GFAP: Glial fibrillary acidic protein; H + E: Hematoxylin and eosin; IgGAQP4: IgG fraction of serum from neuromyelitis optica patients containing AQP4-IgG; IgGCON: IgG fraction of serum from normal subjects; IgGMOG: IgG fraction of serum from neuromyelitis optica patients containing MOG-IgG; IgGMOG(AdsHEK): IgG_MOG_ adsorbed against untransfected HEK cells; IgGMOG(AdsMOG-HEK): IgG_MOG_ adsorbed against MOG-HEK to deplete MOG-IgG; LFB: Luxol fast blue; MBP: Myelin basic protein; MOG: Myelin oligodendrocyte glycoprotein; MOG-IgG2B7: Chimeric anti-mouse MOG recombinant IgG in which the constant mouse regions of the heavy and light chains were substituted with the human IgG_1_ constant regions, C_H_ and C_κ_.; NeuN: Neuronal nuclei; Nfil: Neurofilament; NMO: Neuromyelitis optica.

## Competing interests

The authors declare that they have no competing interests.

## Authors’ contributions

SS designed and carried out the animal experiments, analyzed the data, produced the figures, contributed key ideas and helped draft the manuscript. PW prepared the IgG patient samples and contributed key ideas. GPO produced the recombinant MOG-IgG antibody. JLB produced the recombinant AQP4-IgG antibody and contributed key ideas. AV contributed key ideas and helped to draft the manuscript. MCP participated in the design and coordination of the study and wrote manuscript. All authors read and approved the final manuscript.
